# Bacteria and viruses in the upper respiratory tract of Congolese children with radiologically confirmed pneumonia

**DOI:** 10.1186/s12879-021-06570-1

**Published:** 2021-08-19

**Authors:** Archippe M. Birindwa, Jerry K. Kasereka, Lucia Gonzales-Siles, Shadi Geravandi, Mambo Mwilo, Léonard K. Tudiakwile, Néné L. Mwinja, Balthazar Muhigirwa, Théophile Kashosi, Jeanière T. Manegabe, Elie B. Bugashane, Stay M. Saili, Clement Mungo, Rickard Nordén, Rune Andersson, Susann Skovbjerg

**Affiliations:** 1 Department of Infectious Diseases, Institute of Biomedicine, University of Gothenburg, Gothenburg, Sweden; 2 Panzi Hospital, Bukavu, Democratic Republic of the Congo; 3 Université Evangélique en Afrique, Bukavu, Democratic Republic of the Congo; 4 Department of Clinical Microbiology, Sahlgrenska University Hospital, Region Västra Götaland, Gothenburg, Sweden; 5 CARe – Centre for Antibiotic Resistance Research, Gothenburg University, Gothenburg, Sweden; 6 Hôpital Général de Référence de Panzi, BP: 266, Bukavu, Democratic Republic of the Congo

**Keywords:** Bacteria, Viruses, Nasopharynx, Radiologically confirmed pneumonia, Hospitalised children, Pneumococcal serotypes/serogroups, Democratic Republic of the Congo

## Abstract

**Background:**

Acute pneumonia remains a leading cause of death among children below 5 years of age in the Democratic Republic of the Congo (DR Congo), despite introduction of the 13-valent pneumococcal conjugate vaccine (PCV13) in 2013. Potential pathogens in the nasopharynx of hospitalised children with pneumonia have not been studied previously in DR Congo. Here we compare clinical characteristics, risk factors and nasopharyngeal occurrence of bacteria and viruses between children with severe and non-severe pneumonia.

**Methods:**

Between June 2015 and June 2017, 116 children aged from 2 to 59 months hospitalised due to radiologically confirmed pneumonia at Panzi referral university hospital, Bukavu, Eastern DR Congo were included in the study and sampled from nasopharynx. A multiplex real-time PCR assay for detection of 15 different viruses and 5 bacterial species was performed and another multiplex PCR assay was used for pneumococcal serotype/serogroup determination.

**Results:**

During the study period 85 (73%) of the children with radiologically confirmed pneumonia met the WHO classification criteria of severe pneumonia and 31 (27%) had non-severe pneumonia. The fatality rate was 9.5%. Almost all (87%) children were treated with antibiotics before they were hospitalised, in most cases with amoxicillin (58%) or trimethoprim-sulfamethoxazole (20%). The frequency of potential pathogens in the nasopharynx of the children was high, and any viral or bacterial nucleic acids present at high levels, irrespective of species or type, were significantly associated with severe pneumonia as compared with non-severe cases (52% versus 29%, *p* = 0.032). White blood cell count > 20,000/μL and C-Reactive Protein > 75 mg/dL were associated with severe pneumonia at admission. Fatal outcome was in the multivariable analysis associated with having a congenital disease as an underlying condition. One or more pneumococcal serotypes/serogroups could be identified in 61 patients, and out of all identified serotypes 31/83 (37%) were non-PCV13 serotypes.

**Conclusions:**

The occurrence of any bacteria or any viruses at high levels was associated with severe pneumonia at admission. Children with congenital disorders might need a higher attention when having symptoms of acute respiratory infection, as developed pneumonia could lead to fatal outcome.

## Background

Acute pneumonia remains a leading cause of childhood morbidity and mortality worldwide [1] although the estimated number of pneumonia episodes in young children decreased from 180 million in 2000 to 140 million in 2015 [2]. Pneumonia caused deaths in 0.9 million children below five years worldwide in 2015 [3] and is a leading cause of death in young children in the Democratic Republic of the Congo (DR Congo) [2]. The country has the fourth highest absolute number of pneumonia-related deaths worldwide and the second highest in Africa for children under 5 years of age [2]. The incidence of clinical childhood pneumonia decreased in the DR Congo from over 400 cases per 1000 children and year in 2000 to less than 300 in 2015 [2]. However, acute lower respiratory infections (ALRI) caused 20% of deaths among children aged between 1 and 59 months in 2017, with a death rate of 9.4 per 1000 live births [4].

Approximately 60% of Congolese children with acute pneumonia are treated by inappropriate care providers, such as traditional practitioners, vendors of medicines, relatives or friends [5] and up to 40% do not have access to the correct antibiotic treatment [6]. Moreover, abuse of antibiotics is abundant due to self-medication or consulting a non-appropriate health care provider, as well as over-prescription by clinicians [7, 8].

The burden of bacterial pneumonia has been reduced significantly in children worldwide after the introduction of both conjugate *Haemophilus influenzae* type b and pneumococcal conjugate vaccines (PCVs) into the routine childhood immunisation programs [9]. However, *Streptococcus pneumoniae* (the pneumococcus) still remains an important cause of pneumonia in many settings [1, 10]. The virulence of *S. pneumoniae* is largely due to its polysaccharide capsule which protects it from phagocytosis and elimination by the host immune system and is the basis for epidemiological categorization of the pneumococci into more than 100 different serotypes[11, 12]. In the Eastern DR Congo the 13-valent pneumococcal conjugate vaccine (PCV13) was introduced in 2013.

Nowadays, with the more frequent use of molecular diagnostics, respiratory viruses are increasingly detected among children with acute respiratory disease [1, 13]. Respiratory syncytial virus (RSV) has been reported as the most common virus in children below one year of age hospitalized with ALRI [14, 15]; however, other respiratory viruses, such as influenza virus and enterovirus are frequently detected as well [1, 14–16]. However, discerning the aetiology of childhood pneumonia is complex because respiratory pathogens, including a wide range of bacteria and viruses, are frequently found in healthy children [14, 15].

Increased knowledge surrounding the clinical presentation and pathogens present in children with acute pneumonia may improve future clinical management and prevention of the infection, not least in low-income settings such as DR Congo which not only has a high disease burden [2] but also high rates of antibiotic resistance in the pneumococci colonizing young children [17]. In DR Congo the antibiotic treatments available are entirely empiric due to insufficient microbiological diagnostics and, to our knowledge no studies exist which have assessed the burden of potential pathogens in children hospitalized due to acute respiratory infection. Our objective was to describe clinical characteristics and risk factors and to determine the occurrence of bacteria and viruses in the nasopharynx of hospitalised children with pneumonia in the Eastern DR Congo. We also aimed to relate these findings to the severity of the disease and outcomes.

## Methods

### Study site

The study was conducted at Panzi Hospital in Bukavu town in the South Kivu region of DR Congo, which served a population of 470,000 inhabitants including 89,000 children below 5 years in 2017. The hospital is the referral university hospital for three general hospitals, four health centres and eight dispensaries located in the catchment population area, but the hospital also receives patients from outside this area. Panzi Hospital has 69 paediatric beds with an emergency ward which has 12 beds for acute and severe cases requiring nasal oxygen therapy. The department has two trained paediatricians and four resident doctors. The hospital has a radiology department run by a trained senior specialist in radiology.

### Study patients

Out of a total of 2322 children between 2 months and 5 years of age treated for any disease at the Emergency and admissions Unit, Paediatric Department, between June 2015 and June 2017, 184 were diagnosed with acute lower respiratory infections. Of these, 116 cases had pneumonia that was radiologically confirmed and these children were thus included in the study. Among the 116 included children 80 came from the hospital catchment population area whereas 36 children came from other locations, in most cases nearby rural areas. Children admitted for severe malaria with pneumonia as complication were not included due to difficulties differentiating infection from pulmonary oedema. The parents or guardians of five HIV-positive children declined participation in the study and were thus not included while another three HIV-positive children were included. Four patients referred from health centres to Panzi Hospital had X-ray confirmed pneumonia but were not treated at the hospital because of the cost of care that was regarded as too high and were therefore not included. The direct cost of 5 days’ treatment for pneumonia at Panzi Hospital could amount to 150 US dollars.

Data were collected on age, sex, dates of admission, duration of hospital care, as well as underlying conditions, including malnutrition, HIV, sickle cell disease, cerebral palsy, post-neonatal anoxia and congenital diseases. The WHO child growth standards were used to evaluate the nutritional status of children by computing the Z-scores of weight-for-age and height-for-age [18].

Patients were classified according to the revised WHO classification for pneumonia. This encompasses non-severe pneumonia with fast breathing and/or chest indrawings. Fast breathing was present when the respiratory rate was ≥ 50 breaths per minute for infants between 2 and 12 months of age and ≥ 40 breaths per minute for children between 12 months and 5 years of age [18]. Children were considered to have severe pneumonia when there were any additional danger signs (e.g. not able to drink or breastfeed, persistent vomiting, convulsions, lethargy or loss of consciousness, stridor in a calm child or severe malnutrition) [19].

Saturation of peripheral oxygen (SpO_2_) was measured on admission by using a pulse oximeter (Handheld Pulse Oximeter 3.7vx1, Li-ion, Model No.AH-M1, Acare Technology Co., Ltd, London, UK). Venous blood samples were collected for measurement of white cell count and C-reactive protein. A nasopharyngeal swab was taken from all children in the study (see below). After a physical examination, radiologist reports and blood test analyses, all admissions were validated by the paediatrician. Lung auscultations were considered as abnormal if crackles and/or rhonchi were present.

Data on pre-hospitalisation use of antibiotics were obtained from the parent or guardian asked if any antibiotics were given to the child during the week in which the symptoms of pneumonia began, or from the referral note from any first-level health care facility. The use of antibiotics and nasal oxygen therapy during hospitalisation was also recorded.

Patient outcomes were grouped into the following three categories: survived without complications, survived with complications (e.g. pneumothorax, empyema, or pleural effusion) and death. The group surviving without complications included patients discharged with improvements and who had switched to oral antibiotic treatment. This group included those discharged against medical advice but who were receiving oral antibiotic treatment.

The study was approved by the Ethics Committee at the Université Catholique de Bukavu, DR Congo (UCB/CIE/NC/22/2014), and the Regional Ethics Committee in Gothenburg, Sweden (Dnr: 504-16). The study was further approved by the South-Kivu province Health Organisation (065/CD/DPS-SK/2015) and by the Director of Panzi Hospital.

### Assessment of chest X-ray

The diagnosis of pneumonia was confirmed by X-ray (Siemens-Elema AB, Solna Sweden) with anteroposterior and lateral chest radiographs obtained from all included children. The images were first analysed by the radiologist at Panzi Hospital who was trained in the standard interpretation of chest radiographs for the diagnosis of childhood pneumonia. The second and final validation was performed by a senior specialist radiologist according to the WHO standard interpretation of chest radiographs for the diagnosis of pneumonia in children. Pneumonia was confirmed by the presence of consolidation, infiltrates or effusion and these results were considered as chest X-ray confirmed pneumonia [20]. Pleural effusion was present when there were more than 1 cm fluid in the pleural space between the lung and chest wall [20].

### Nasopharyngeal specimen collection

A nasopharyngeal sample was collected from all included children and transported to the Clinical Laboratory at Panzi Hospital for pneumococcal culture as previously described [17]. The nasopharyngeal samples were thereafter stored frozen at − 20 °C before shipment to the Department of Infectious Diseases, University of Gothenburg, Gothenburg, Sweden, where they were stored at − 80 °C prior to the molecular analyses (see below).

### Bacterial and viral nucleic acid detection

Nucleic acids were extracted from 200 µL of the nasopharyngeal sample as described earlier [21]. Eluted nucleic acids were stored at − 20 °C until further analysis.

A multiplex real-time PCR assay capable of detecting 20 different viruses and bacterial species (adenovirus, bocavirus, coronavirus 229E, HKU1, NL63 and OC43, enterovirus, influenza virus A and B, human metapneumovirus, parainfluenza 1–3, rhinovirus, RSV, *Bordetella pertussis*, *Chlamydia pneumoniae*, *H. influenzae*, *Mycoplasma pneumoniae* and *S. pneumoniae*) was performed as previously described [22]. A Cycle Threshold (Ct)-value < 40 was classified a positive result with values of Ct < 30 indicating high levels of nucleic acids. Pneumococci were identified by *lytA* gene detection in nasopharyngeal samples. However, an additional three samples were considered positive for pneumococci consequent to *cpsA* gene detection (Ct < 40) in the serotype assay described below.

### Pneumococcal serotyping

A multiplex real-time PCR assay capable of detecting 40 different pneumococcal serotypes/serogroups in addition to the *cpsA* gene was used according to a previously published protocol [23].

### Data management and statistical analysis

Descriptive analysis was performed using the SPSS package (version 24.0) for logistic regression analysis of the relationship between nucleic acid identification and symptoms, medical conditions or outcome. Differences in baseline data and clinical factors were analysed with Pearson’s chi-square test, and medians were compared with Mann–Whitney U test. The proportions of detected bacteria and viruses in severe and non-severe pneumonia cases were compared by Pearson’s chi-square or Fisher’s exact test (if n < 5). Potential variables associated with differences between fatal and improving cases were assessed by odds ratios (OR) with 95% CI and were tested by univariable analysis with the Pearson’s chi-square or Fisher’s exact test (if n < 5). Associations to fatal outcome with *p* < 0.2 were re-analysed by multivariable analysis. A *p-*value of < 0.05 was considered statistically significant.

## Results

### Characteristics of the included children

Two years after the introduction of PCV13 in the infant immunization program in the Eastern DR Congo, 125/2322 (5.4%) of all children admitted to Panzi Hospital, DR Congo were diagnosed with radiologically confirmed pneumonia. Of these children, 116 were included in the present study (median age 12 months). Ninety-five (82%) of the included children came from Bukavu town while 21 came from rural areas (Table 1).

**Table 1:** Clinical characteristics of the children hospitalised due to severe versus non-severe pneumonia at Panzi Hospital, Bukavu, DR Congo between June 2015 and June 2017 (*n* = 116)

Factors		All cases *n* = 116 (%)	Severe pneumonia *n* = 85 (%)	Non-severe pneumonia *n* = 31 (%)	Univariable analysis *p*-value
Location of residence	Bukavu urban area^1^	95 (82)	69 (81)	26 (84)	0.73
	Bukavu rural area^1^	21 (18)	16 (19)	5 (16)	0.73
Sex	Boys	61 (53)	43 (51)	18 (58)	0.47
	Girls	55 (47)	42 (49)	13 (42)	0.47
Age in months	Median [IQR]^2^	12 [8–36]	13 [8–36]	12 [9–36]	0.98
	≤ 6	26 (22)	10 (12)	6 (19)	0.29
	> 6–12	32 (28)	30 (35)	12 (39)	0.73
	> 12–24	23 (20)	19 (22)	4 (13)	0.26
	> 24–36	14 (12)	12 (14)	2 (7)	0.27
	> 36–60	21 (18)	14 (16)	7 (23)	0.45
Undernutrition^3^		11 (9)	10 (12)	1 (3)	0.19
Other diseases^4^		9 (8)	8 (9)	1 (3)	0.29
PCV13 vaccination^5^	(2 or 3 doses)	78 (67)	55 (65)	23 (74)	0.33
Gastro-intestinal symptoms^6^		30 (26)	21 (25)	9 (29)	0.63
White cells count^7^ (cells/μL)	Median [IQR]	16 [14–20]	17 [14–20]	16 [13–17]	0.018
	4000–15,000	48 (41)	29 (34)	9 (29)	0.60
	> 15,000–20,000	54 (47)	35 (41)	19 (61)	0.58
	> 20,000	23 (20)	21 (25)	2 (7)	0.043
C-Reactive Protein^8^ (mg/dL)	Median [IQR]	64 [28–64]	78 [24–84]	23 [22–69]	0.0004
	< 25	46 (40)	25 (29)	21 (68)	0.0003
	25–75	21 (18)	12 (14)	9 (29)	0.07
	> 75	49 (42)	48 (56)	1 (3)	0.0004
Pre-hospitalisation antibiotic treatment	No antibiotic	15 (13)	6 (7)	9 (29)	0.003
	Amoxicillin, ampicillin or phenoxymethylpenicillin	68 (59)	49 (58)	19 (61)	0.72
	Trimethoprim-sulfamethoxazole	23 (20)	21 (25)	2 (6)	0.043
	*Other antibiotics*^9^	10 (9)	9 (11)	1 (3)	0.23
Antibiotic treatment during hospital stay	Ampicillin and gentamicin	46 (40)	25 (29)	4 (13)	0.07
	Ceftriaxone and gentamicin	62 (53)	53 (62)	9 (29)	0.002
	Others^10^	8 (7)	7 (8)	1 (3)	0.36
Nasal oxygen therapy		100 (86)	79 (93)	21 (68)	0.001
Duration of hospital stay (days)	Median [IQR]	5 [5–8]	5 [5–8]	5 [4, 5]	0.0001
	1–5	70 (60)	44 (52)	26 (84)	0.003
	> 5–10 days	38 (33)	34 (40)	4 (13)	0.009
	> 10 days	8 (7)	7 (8)	1 (3)	0.36
Outcome	Improved	104 (90)	74 (87)	30 (97)	0.16
	Dead	11 (9)	10 (12)	1 (3)	0.19
	Complications	1 (1)	1 (1)	0 (0)	-

^1^Bukavu urban area (n = 95): Ibanda area, n = 80; Kadutu area, n = 13; and Bagira, n = 2

^2^*IQR* interquartile range

^3^Undernutrition defined as weight for age or weight for height as a Z score ≤ -2 standard deviations, determined by ENA for smart software 2011

^4^Other diseases (Congenital and others): sickle cell disease (n = 2), congenital cardiac disorder (n = 1), cerebral palsy (n = 2), Down syndrome (n = 1), and HIV (n = 3)

^5^PCV13 = the 13-valent conjugate pneumococcal vaccine

^6^Gastro-intestinal symptoms: diarrhoea or vomiting

^7^The normal range of White cells count is 4000–10,000 cells/μL

^8^The normal value for C-Reactive Protein is < 10 mg/dL

^9^Other antibiotics (pre-hospitalisation antibiotic treatment): erythromycin (n = 5), ciprofloxacin (n = 3) or cloxacillin (n = 2)

^10^Others (antibiotic treatment during hospital stay): ciprofloxacin (n = 3) and cloxacillin (n = 5)

Almost all the children 101 (87%) were treated with antibiotics before hospital admission, according to the parent or guardian (n = 39) or the referral notes (n = 62). The most common pre-hospitalisation antibiotics were either amoxicillin/ampicillin/phenoxymethylpenicillin or trimethoprim-sulfamethoxazole (Table 1). Two thirds (67%) of the children had received two or three doses of PCV13.

The main signs and symptoms noted during the physical examination at admission were fever or recent history of fever (96%), abnormal lung auscultation (99%), cough or recent history of cough (100%), and rapid or difficult breathing (94%). Malnutrition was present in 11 (9.5%) of the patients. During hospital stay 53% were treated with ceftriaxone and gentamicin whilst 40% received ampicillin and gentamicin (Table 1).

### Differences in clinical characteristics between children with severe and non-severe pneumonia

According to the WHO pneumonia classification, 85/116 (73%) of the children met the criteria for having severe pneumonia at admission whereas 31 (27%) had non-severe pneumonia. Between the two groups, there was no statistically significant difference in sex, age, living area or immunisation status, fever, cough or abnormal auscultation (data not shown). However, a white cell count of over 20,000/μL was significantly associated with severe pneumonia (OR 4.75; 95%CI 1.0–21.6, *p* = 0.043) (Table 1). CRP levels above 75 mg/dL were also significantly associated with severe pneumonia at admission (OR 38.9; 95% CI 5.1–299, *p* = 0.0004) while CRP levels below 25 mg/dL were negatively associated with severe disease (OR 0.19; 95% CI 0.08–0.48, *p* = 0.0003) (Table 1). The median CRP level was much higher in children with severe pneumonia at admission as compared with the non-severe pneumonia cases while the difference in median concentration of the white cells were small between the two groups (Table 1). As expected, nasal oxygen therapy was more often used in children with severe pneumonia as compared to children with less severe disease (Table 1). Overall, pre-hospitalisation antibiotic treatment was more common among children with severe pneumonia than in children with non-severe disease. While penicillin and the penicillin derivatives amoxicillin or ampicillin were equally common in the two groups, trimethoprim-sulfamethoxazole was more commonly used in children with severe pneumonia (OR 4.75; 95%CI 1.04–21.6 as above) (Table 1). During hospital stay, ceftriaxone combined with gentamicin was more frequently used to treat the severe pneumonia cases than the children with non-severe disease (Table 1).

### Bacteria and viruses found in the nasopharyngeal secretions

From only one nasopharyngeal sample pneumococci could be isolated by culture. However, by real-time PCR, performed directly on the nasopharyngeal sample, pneumococci could be detected in almost all children (96%), whereas *H. influenzae* was detected in 54% and *B. pertussis* in 10% (Table 2). When employing a more stringent cut-off level (Ct < 30), bacteria were found in 62% of the samples; *S. pneumonia*e in 53% of the cases, and *H. influenzae* in 20% (Table 2).

**Table 2:** Real-time PCR using the cycle threshold (Ct) levels < 40 and Ct < 30, respectively, for pathogen detection in nasopharyngeal secretions from hospitalised children 2 to 59 months of age with severe or non-severe radiologically confirmed pneumonia

Pathogens	Number of cases (%)								
	Real-time PCR detection level Ct < 30				Real-time PCR detection level Ct < 40				
	All pneumonia cases n = 116	Severe pneumonia n = 85	Non-severe pneumonia n = 31	*p*-value	All pneumonia cases n = 116	Severe pneumonia n = 85	Non-severe pneumonia n = 31	*p*-value	*Ct* value, median (severe/non-severe pneumonia)
Bacteria									
*Streptococcus pneumoniae*	61 (53)	44 (52)	17 (55)	0.76	111 (96)	81 (95)	30 (97)	0.72	30.8/30.4
*Haemophilus influenzae*	23 (20)	18 (21)	5 (16)	0.54	63 (54)	50 (59)	13 (42)	0.10	32.2/30.7
*Bordetella pertussis*	1 (1)	1 (1)	0 (0)	–	12 (10)	9 (11)	3 (9)	0.88	37.8/39.1
*Chlamydia pneumoniae*	0 (0)	0 (0)	0 (0)	–	5 (4)	3 (4)	2 (6)	0.49	34.7/34.9
*Mycoplasma pneumoniae*	0 (0)	0 (0)	0 (0)	–	4 (3)	3 (4)	1 (3)	0.93	31.5/36.2
Any bacteria	**72 (62)**	**59 (69)**	**13 (42)**	**0.008**	112 (96)	81 (95)	30 (97)	0.72	–
Viruses									
Rhinovirus	34 (29)	27 (32)	7 (23)	0.33	85(73)	64(75)	21(78)	0.41	31.8/32.9
Enterovirus	2 (2)	1 (1)	1 (3)	0.47	20 (17)	16 (19)	4 (13)	0.45	36.8/34.7
Adenovirus	5 (4)	4 (5)	1 (3)	0.72	13 (11)	9 (11)	4 (13)	0.72	34.0/–
Bocavirus	5 (4)	4 (5)	1 (3.2)	0.72	12 (10)	8 (9)	4 (12.9)	0.58	31.1/37.2
Parainfluenza virus	3 (3)	3 (4)	0 (0)	–	9 (8)	9 (11)	0 (0)	–	31.5/–
RSV	5 (4)	4 (5)	1 (3)	0.72	8 (7)	7 (8)	1 (3)	0.36	27.9/28.2
Coronavirus	1 (1)	1 (1)	0 (0)	–	8 (7)	6 (7)	2 (6)	0.90	33.6/34.2
Influenza A virus	1 (1)	1 (1)	0 (0)	–	3 (3)	1 (1)	2 (6)	0.15	–
Influenza B virus	0 (0)	0 (0)	0 (0)	–	1 (1)	1 (1)	0 (0)	–	–
Human metapneumovirus	0 (0)	0 (0)	0 (0)	–	1 (1)	1 (1)	0 (0)	–	–
Any virus	**53 (47)**	**44 (52)**	**9 (29)**	**0.032**	106 (91)	78 (92)	28 (90)	0.80	–
Any bacteria and virus	29 (25)	24 (28)	5 (16)	0.18	104 (90)	76 (89)	28 (90)	0.88	–

Bold indicates result with **p**-value < 0.05 that was considered statistically significant

The most frequently detected virus was rhinovirus (73%), followed by enterovirus (17%), while RSV was found in only 7% of the cases and influenza virus was rare (Table 2). When only high levels of viral nucleic acids were considered (Ct-value < 30), rhinovirus was found in 29% of the cases, and only a few samples were positive for RSV and influenza virus, respectively (Table 2). No statistically significant differences were found in the frequencies nor in the Ct median levels for any of the detected bacterial species or viruses between severe and non-severe pneumonia cases. However, high levels (i.e. Ct-value < 30) of any virus (OR 2.63; *p*-value = 0.032) or any bacteria (OR 3.14; *p*-value = 0.008) present in the sample, regardless of type or species was significantly associated with severe pneumonia (Table 2).

### Serotypes/serogroups of the pneumococci detected by real-time PCR

**Fig. 1:**
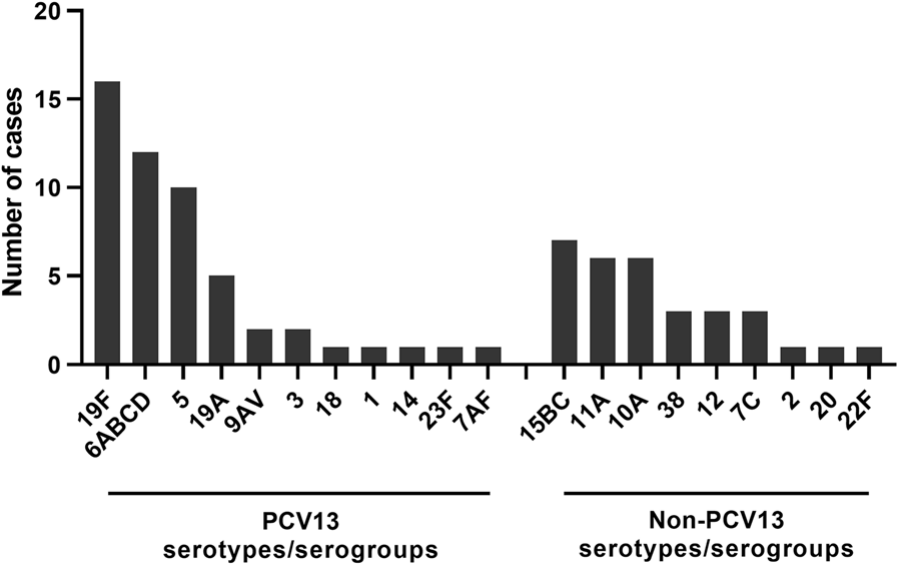
Identification of 83 pneumococcal serotypes by real-time PCR in nasopharyngeal secretions from 61 children out of 116 hospitalised children with radiologic confirmed pneumonia. The method could not distinguish between the 6ABCD serotypes, of which only 6A and 6B are in PCV13. Nor could the method separate 9AV, of which only 9V is in PCV13

Out of the 111 nasopharyngeal samples that were positive for pneumococci (Ct < 40), 83 serotypes/serogroups could be identified in samples from 61 children while no serotype could be detected in secretions from the remaining 50 children. Two different serotypes/groups could be identified in 11 patients, while three children had three, one child had four, and another child had five different serotypes/groups. Among the 83 identified serotypes, 52 (63%) belong to the PCV13 serotypes/serogroups, while the remaining 31 (37%) are non-PCV13 serotypes/serogroups. The serotypes/groups most frequently detected were 19F, 6ABCD and 5 while the predominant non-PCV13 serotypes were 15BC, 10A and 11A (Fig. 1). Two samples were found to be negative for the *cps*A pneumococcal capsule gene but were strongly positive for the *lytA* gene (Ct < 30), indicating non-encapsulated non-typeable pneumococci.

### Fatal outcome

The overall case fatality rate among the hospitalized children with radiological confirmed pneumonia was 9.5% (11 children). Five of these children died within 48 h after hospital admission and another four died after two to five days**.** Fatal outcome was significantly associated with having a congenital disease as an underling condition (Table 3). Univariable analysis also showed an association between fatal outcome and high nucleic acid levels (Ct < 30) of pneumococci or RSV in the upper respiratory tract of the children, but this could not be confirmed in the multivariable analysis (Table 3). The two RSV-positive cases with fatal outcome were also positive for pneumococci. Two different pneumococcal serotypes could be determined in the nasopharyngeal secretions from the children with fatal outcome; serotype 19F in four cases and 15BC in one case. In the remaining five children with pneumococci detected, no serotype could be identified.

**Table 3:** Clinical characteristics, risk factors and microbiological findings among the children with fatal outcome compared to improved children hospitalized due to radiologically confirmed pneumonia (n = 116)

Clinical characteristics/risk factors/microbiological findings		Outcome				
		Dead (*n* = 11)	Improved (*n* = 104)	OR (95% CI)	*p*-value	Adjusted *p*-value^4^
In hospital antibiotic treatment	Ceftriaxone + gentamicin	6 (55)	56 (54)	1.02 (0.29–3.58)	0.96	
	Ampicillin + gentamicin	5 (45)	41 (39)	1.28 (0.36–4.47)	0.69	
Nasal oxygen^1^		10 (91)	89 (86)	1.68 (0.20–14.14)	0.63	
Underlying conditions	Congenital diseases^2^	**4 (36)**	**5 (5)**	**15.84 (3.22–77.86)**	**0.0007**	**0.004**
	Malnutrition^3^	2 (18)	9 (9)	2.34 (0.43–12.55)	0.31	
Positive real-time PCR (Ct-values < 30)	*Streptococcus pneumoniae*	**10 (91)**	**51 (49)**	**10.39 (1.28–84.12)**	**0.028**	**0.81**
	*Haemophilus influenzae*	3 (27)	20 (19)	1.57 (0.38–6.47)	0.52	
	Rhinovirus	3 (27)	31 (30)	0.88 (0.21–3.55)	0.86	
	RSV	**2 (18)**	**3 (3)**	**7.48 (1.10–50.76)**	**0.039**	**0.99**
	At least one bacteria + at least one virus	3 (27)	24 (23)	1.25 (0.30–5.08)	0.75	

Bold indicates result with *p*-value < 0.05 that was considered statistically significant

^1^Nasal oxygen treatment = 0.5–2 L/min

^2^Congenital diseases = sickle cell disease (n = 2), congenital cardiac disorder (n = 1), cerebral palsy (n = 2), Down syndrome (n = 1) and HIV (n = 3)

^3^Malnutrition defined as weight for age or weight for height as a Z score ≤ − 2 standard deviations, determined by ENA for smart software 2011

^4^All factors with *p* < 0.2 in the univariabel analysis were included in the multivariable analysis: Congenital diseases, RSV and *Streptococcus pneumoniae* detected with Ct-value < 30

## Discussion

This is the first study on hospitalised children with radiologically confirmed pneumonia in DR Congo in the PCV13 post-vaccine era. It includes both clinical and microbiological aspects. A majority (73%) of the children were diagnosed as having severe pneumonia. The most common symptoms for both severe and non-severe pneumonia cases were fever, cough or abnormal auscultation, similar to that described in a Vietnamese [24] and a Tanzanian study [25]. High white cell counts (> 20,000 cells/μL) and high CRP levels (> 75 mg/dL) were associated with severe pneumonia at admission. Similar findings of elevated white cell counts in hospitalised children with pneumonia were reported from Senegal [26], as well as high CRP levels (> 80 mg/dL) also being associated with radiologically confirmed pneumonia in Tanzanian children [15].

The case fatality rate in our study was 9.5%, similar to findings from a Tanzanian district hospital (11%) [25] but higher than those reported from hospitalised children in Cambodia (3.2%) [27]. Up to 80% of deaths in children with severe respiratory infections may occur outside hospitals in low-income countries [28]. It is possible that some children first receive self-medication by parents or guardians, first seeking care at a private pharmacy, traditional practitioner or at any other non-appropriate health care provider before attending professional health care facilities [5, 8, 28–30]. This is supported by the fact that less than half (42%) of under-five children with suspected pneumonia in DR Congo were found to have been treated by a trained health care provider [8].

Eighty-seven percent of the children included in our study received pre-treatment with antibiotics, in most cases amoxicillin, ampicillin or phenoxymethylpenicillin (59%) followed by trimethoprim-sulfamethoxazole (20%). In 34% of the cases, information about pre-hospitalisation medication was obtained from patient or guardian reports only and no information was obtained on duration of the treatment. Earlier reports show that parent history of antibiotic treatment in children is not always reliable [31–33]. Bioassays for detection of antibiotic activity in urine or serum may be of value for assessment of pre-hospitalisation antibiotic use [34–36] but these were not performed in the present study. However, from earlier studies it is known that pre-hospitalisation medication is very common in low-income countries, especially when there is limited health care access and high availability of antibiotics [5, 8]. Fifteen years ago, it was found that trimethoprim-sulfamethoxazole and amoxicillin were frequently used in self-medication in Congolese children having cough with or without fever [5]. We have recently showed a high level of resistance against trimethoprim-sulfamethoxazole in pneumococci colonizing children in the general population in this area of DR Congo [17] and similar results were shown for healthy children in northern Tanzania [37]. Here we found that pre-hospitalisation medication by the oral broad spectrum antibiotic trimethoprim-sulfamethoxazole was more common in children with severe pneumonia than in children with non-severe disease. However, since no data was collected on the duration of symptoms or treatment, the clinical impact of this result should be interpreted with caution.

Recently the DR Congo introduced clinical guidelines for the management of pneumonia. However, the antibiotic management of severe pneumonia was not included. For optimizing antibiotic treatment regimens antimicrobial resistance testing and surveillance are needed, especially considering the high level of resistance to commonly used antibiotics in the country [17]. In the present study, ceftriaxone and gentamicin were the main antibiotics used for treating severe pneumonia. This corroborates the findings of a study in Senegal where more than 55% of pneumonia cases were treated with ceftriaxone [26, 38].

The low isolation rate of live pneumococcus (0.9%) by culture in the present study may be explained by the high frequency of pre-treatment with antibiotics. The pneumococcal isolation rate was higher (21%) among the 794 Congolese children from the general population included in our previous study; also, 7% of these children had been treated with antibiotics in the last month according to their parents or guardians [17]. If carried out before specimen collection, this is known to reduce the culture isolation rate of bacteria but has less impact on the detection rate by PCR [36, 39]. Here we detected pneumococci by PCR in almost all (96%) nasopharyngeal secretions and *H. influenzae* in 54%. This result corroborates findings from Zanzibar, Tanzania, in which pneumococci were found in 87% and *H. influenzae* in 77% of febrile children [15]. When employing a more stringent cut-off level, (i.e. Ct-value < 30) in which only high amounts of microbial nucleic acids are identified, we found *S. pneumoniae* in 53%, and *H. influenzae* in 20% of the cases. We could not show any significant differences between the age groups and presence of pathogens as reported in other studies [40, 41] nor an association between severity of disease and specific viruses or bacterial species as identified in the study. However, high levels of bacterial or viral nucleic acids in the nasopharynx (irrespective of the species or types) were associated with more severe pneumonia at admission, compared to less severe cases. This supports the importance of not only bacteria but also viruses in the development of severe pneumonia.

Surprisingly, our detection rate of 7% RSV was much lower than the 31% reported in a multicentre study in Africa and Asia [14]. This discrepancy might be explained by the geographic variability of pathogens [14] or to possible degradation of nucleic acids during transport and storage of the samples eventually affecting the level of detection. However, although only a few cases were detected, RSV was more prevalent in children that died, also having high nucleic acid levels of pneumococci. In the two fatal RSV-positive cases, pneumococci were also detected, supporting the evidence of RSV being associated with pneumococci in critical cases.

The frequent co-occurrence of viruses and bacteria in childhood pneumonia [14] has been associated with disease severity [10, 14]. Infection with RSV facilitates colonisation with bacteria such as *S. pneumoniae* and *H. influenzae* in the nasopharynx of young children [42]. In addition, invasive pneumococcal infection increases during the seasonal peak of various respiratory viruses, including influenza virus and RSV [14].

Since we did not include a hospital based control group, and most of these pathogens are also prevalent in healthy children, it was not possible to discern the aetiology of the pneumonia cases in the present study, not even when only microbial nucleic acids in large amounts were considered [14, 15, 43].

PCV13 serotypes/serogroups were more commonly identified than non-PCV13 serotypes/groups (63% versus 37%). This was in contrast to a study performed in Mozambique, in which half of the identified pneumococcal serotypes were included in the vaccine [44]. However, we may have under-estimated the frequency of non-PCV13 serotypes. This is because our method could neither distinguish between the 6ABCD serotypes, (of which only 6A and 6B are in PCV13), or between 9A and 9V (of which only 9V is in PCV13). Moreover, the assay measures all the serotypes included in the pneumococcal vaccines but few of the additional ones. This suggests that, because those 50 samples were positive for pneumococci according to our PCR assay, but were negative for the serotypes included in the detection panel, they may have contained non-vaccine serotype pneumococci. Thus, non-vaccine serotypes were most likely much more prevalent than we detected here. As seen in other studies non-vaccine serotypes are continually emerging after the introduction of PCV13 [45, 46]. Here, serotype 19F was the most frequent PCV13 serotype, whereas serotypes 15BC, 11A and 10A were the most prevalent non-PCV13 serotypes. This was similar to our findings in the general child population in the same area [17]. In this way, similar information about serotype distribution was obtained from healthy children as from children with pneumonia requiring hospital stay.

Our study had several limitations. First, obtained swab specimens were stored at − 20 °C in DR Congo before being transported to Sweden for storage at − 80 °C prior to the molecular analyses. It cannot be excluded that bacterial or viral nucleic acids were degraded during transport and storage, possibly affecting the detection levels of DNA and RNA. Nonetheless, rhinovirus RNA and pneumococcal DNA were detected in the majority of samples indicating sufficient storage conditions of both types of nucleic acid species. Second, in addition to referral notes from primary health care facilities, data on pre-hospitalisation antibiotic treatment were collected by questioning the parents or guardians, which might lead to unreliable or incomplete data. No data were collected on duration of symptoms or treatment before coming to the hospital, which might have an impact of the outcome and severity of the disease. Finally, the study was performed at a referral university hospital where treatment was associated with an unneglectable cost, which might affect the representativeness in the area.

Strengths of the study included the broad range of potential pathogens that we were able to detect in the children, and presentation of pneumococcal serotype data, which are very scarce in this resource-limited setting. We included only radiologically confirmed pneumonia cases, since clinical diagnosis of childhood pneumonia is difficult and unspecific, which strengthen the validity of the results from the study.

## Conclusions

Any high bacterial or viral nucleic acid levels were more often detected in children having severe pneumonia than in those with non-severe disease. RSV and influenza were rarely detected. White blood cell count > 20,000***/***μL and CRP levels > 75 mg/dL were associated with severe pneumonia at admission while congenital disease was associated with fatal outcome. Out of all identified pneumococcal serotypes/serogroups 37% were not in PCV13.

## Data Availability

The datasets used and analysed during the current study are available from the corresponding author on reasonable request.

## Abbreviations

CARe: Center for Antibiotic Resistance Research, Gothenburg
CDC: Centers for Disease Control and Prevention
CI: Confidence interval
DR Congo: Democratic Republic of the Congo
ENA: Emergency Nutrition Assessment
EUCAST: European committee on antimicrobial susceptibility testing
HIV: Human immunodeficiency virus
OR: Odds ratio
PCV: Pneumococcal conjugate vaccine
PCV13: 13-Valent pneumococcal conjugate vaccine
TMP-SMX: Trimethoprim-sulfamethoxazole
WHO: World Health Organisation
